# Assessing the GRIP of Ventral Hernia Repair: How to Securely Fasten DIS Classified Meshes

**DOI:** 10.3389/fsurg.2017.00078

**Published:** 2018-01-19

**Authors:** Friedrich Kallinowski, F. Harder, D. Gutjahr, R. Raschidi, T. G. Silva, M. Vollmer, Regine Nessel

**Affiliations:** ^1^Klinik für Allgemein-, Visceral- und Transplantationschirurgie, Chirurgische Universitätsklinik Heidelberg, Heidelberg, Germany; ^2^Institut für Biomechanik, Technische Universität Hamburg-Harburg, Hamburg, Germany

**Keywords:** ventral hernia repair, GRIP, bridging, overlap, hernia meshes, fixation, glue, model for ventral hernia repair

## Abstract

Recurrences are frequently observed after ventral hernia repair. Based on clinical data, the mesh–defect area ratio (MDAR) can lead to lower recurrence rates. Using dynamic intermittent strain (DIS) in a pig tissue model, MDAR can be modified to give a measure called grip to better assess the mechanical stability of ventral hernia repair. The focus of this experimental study is to assess the different aspects of mesh overlap (OL) and fixation only in bridging repair of ventral hernias. DIS mimics coughing actions in an *ex vivo* model with the repetition of submaximal impacts delivered *via* a hydraulically driven plastic containment. Tissue derived from pig bellies simulates a ventral hernia with varying defect sizes. MDAR is calculated from the hernia orifice and the mesh OL. Commercially available meshes were strengthened with glue, tacks, and sutures to bridge the defects. The reconstructions are strained with up to 425 dynamic impacts. The grip of each repair is assessed using MDAR modified by the strength of the fixation. The DIS classification is based on bridging of a 5 cm ventral hernia orifice with an OL of 5 cm in a sublay position. The classification discriminates meshes properties upon DIS strain. MDAR is calculated to be 9 under these conditions. Decreasing the OL or increasing the hernia orifice reduces MDAR to numbers below 9. MDAR is modified to reach GRIP. Closure of the peritoneum adds about 4 to the grip given by MDAR. The multiplying factor of a transmural suture or one tack of Securestrap^®^ or Protack^®^ is 0.5 times the number of tacks applied. The multiplier given by a bonding spot of Glubran^®^ is similar to that of an Absorbatack^®^ being 0.33. Plotting the likelihood of a bridging repair to survive more than 400 DIS impacts versus the grip estimated from the factors given above, the grip to be passed for a durable repair is 10 for Parietex Progrip^®^, and Dynamesh Cicat^®^ and 25 for Dynamesh IPOM^®^. Clinical data previously published can be reculculated to assess MDAR and permit an estimation of the grip of the reconstruction. In these recalculations, a correlation between MDAR and long-term recurrence rates is found. A dimensionless number called grip can be calculated. The grip can be modified by fixation in a reproducible way. A higher grip can improve the durability of ventral hernia repair. We believe that a higher grip leads to lower recurrence rates in the clinical setting.

## Introduction

The repair of ventral or incisional hernias frequently fails (1, 2). For laparoscopic repair, an overlap (OL) of 3 better 5 cm is mandatory to reach low recurrence rates (3). Tulloh and de Beaux (4) proposed to study the importance of the mesh:defect area ratio (MDAR) as a predictor of recurrence after ventral hernia repair. Based on the work of Tse et al. (5) and others, they calculated a MDAR threshold of 16 to be exceeded for lowered recurrence rates. Recently, Hauters et al. (6) published a prospectively observed cohort with laparoscopic ventral and incisional hernia repair. This paper reports a strong correlation between MDAR and the recurrence rates observed after more than 5 years.

Mesh–defect area ratio is most probably a measure for the static cohesion. Static cohesion varies with the surface properties of any mesh touching tissues. A precondition for the occurrence of an adhesion is that two bodies touch and the contact surface is loaded by an outer force (7). Any solid objects pressing against each other (but not sliding) will require some threshold of force parallel to the surface of contact in order to overcome static cohesion. Stiction is a *threshold*, not a continuous force. However, MDAR is independent from tissue, mesh, or fixation properties. A biomechanical measure is sought after to account for the interaction of mesh, fixation, surgical procedure, and tissue keeping the mesh in place until healing occurs.

The threshold of a force parallel to the surface characterizing a distinct ventral hernia repair can be examined with dynamic intermittent strain (8–10). Delivering dynamic intermittent strain (DIS) up to 250 mmHg repeatedly in an *ex vivo* model can test the impact of repeated strain similar to jumping, coughing, or vomiting on the stability of ventral hernia repair (8). First results demonstrated a rapid deterioration of ventral hernia repair in the majority of applications tested upon repeated impacts (9). A classification was proposed distinguishing primarily stable, intermediate, and unstable repairs upon DIS testing (9, 10). Since most ventral hernia repairs examined so far deteriorate rapidly upon dynamic intermittent strain ways to reliably stabilize primarily unstable ventral hernia repairs are examined here. It is found that a factor called grip characterizes the threshold to be exceeded in the experimental model if repair techniques are intended to withstand more than 400 coughing actions. The experimentally found grip factor depends on tissue, mesh, and fixation properties. In order to gain further insight into the clinical significance of the grip factor, experimental data acquired here are compared to clinical MDAR data derived from previous publications. The focus of this experimental study is to assess the different aspects of mesh OL and fixation only in bridging repair of ventral hernias. In order to gain insight into prospective clinical data, the HERNIAMED registry was expanded to include MDAR and grip as part of the STRONGHOLD application for participating centers. Future development of DIS testing might include repair techniques with closure of the defect. Progress of DIS testing can be monitored on www.hernia-today.com.

## Materials and Methods

### Experimental Pig Belly Model

A tissue model was used for the application of dynamic intermittent strain (DIS). The *ex vivo* model hydraulically pushes a plastic containment in an aluminum cylinder (8). The new version of the model permits control of the length of the pressure plateau during the descent from the peak flow (11). The destabilization increases by about 10% with a prolongation of the pressure plateau by about 0.1 s. For this paper, the length of the pressure plateau ranged between 0 and 0.1 s yielding similar dislocation rates on both machine versions for a given condition (data on www.hernia-today.com). Commercially available full thickness pig bellies were selected as described earlier (9, 10). Since the tissue elasticity markedly influences the likelihood of dislocation medium range pig bellies were selected (9). An elastic membrane replaces skin and subcutaneous tissue which result in uncontrollable viscoelasticity (EPDM 90 shore, Kuhn & Kaiser, Erndtebrück, Germany). The membrane was punched with a central defect of 5 or 7.5 cm, respectively. As a lubricant, commercially available Vaseline^®^ (Sanofi-Aventis, Frankfurt/M., Germany) was used as described previously (8–10).

### Meshes and Fixation Methods

As meshes, Dynamesh CiCAT^®^ and Dynamesh IPOM^®^ (FEG Textiltechnik, Aachen, Germany) and Parietex Progrip^®^ (Medtronic Deutschland, Meerbusch, Germany) were investigated. As sutures, Monomax 4 metric (B. Braun, Melsungen, Germany) and Maxon^®^ 4 metric, Novafil^®^ 3 metric with the V-20 taper needle or Surgipro^®^ 4 metric with the GS-21 taper needle (Medtronic Deutschland, Meerbusch, Germany) were used. In previous DIS tests on the suture strength, no difference was detected in the suture retention in pig tissue when stitches were applied transmurally with an U-shaped stitch 0.8 cm wide. Three different fixation devices were tested: Securestrap^®^ (Ethicon, Hamburg, Germany), AbsorbaTack^®^ and ProTack^®^ (Medtronic Deutschland, Meerbusch, Germany). As bonding fixation, Glubran^®^ glue was supplied by Dahlhausen (Köln, Germany). As fixation method, single crowns were tested with half, quarter and eighth tooth peaked as described before (8–10). Perpendicular positions were chosen when only four fixations points were used. Since rotation of the fixation spots influences the strength of the fixation, care was taken to always include the linea alba (data not shown). The position of the gluing spots was chosen accordingly. The size of the gluing spots was normalized as 0.8 cm in diameter as described previously (10).

### Calculation of the Grip Factor

**Figure 1:**
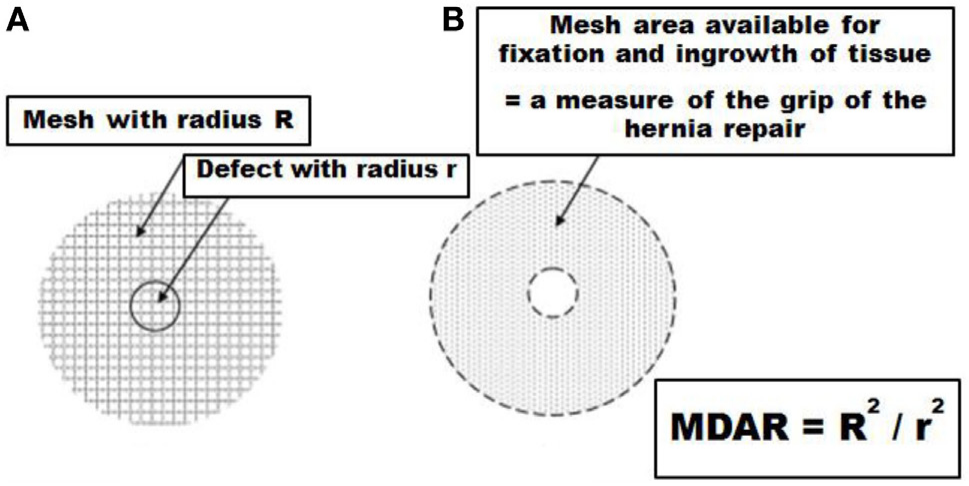
Description of the biomechanical background of the GRIP calculation based on the area of the mesh and the diameter of the hernia orifice [mesh–defect area ratio (MDAR), as given by Tulloh and de Beaux (4)] **(A)**. The area available for MDAR enables fixation and tissue ingrowth **(B)**. The formula as given by Tulloh and de Beaux (4) was modified in this manuscript to include the static friction added by sutures, tacks, and glue to assess the grip of the hernia repair.

The grip factor is directly related to the area of the mesh and the diameter of the hernia orifice (MDAR). According to Tulloh and de Beaux (4) the ratio of the respective mesh and hernia defect areas are a measure of the static friction given by the mesh area available for fixation and ingrowth of tissue (Figures 1A,B). Since the result of this calculation is a measure without dimension, it is termed MDAR to easily name the number yielded by the ratio R*R:r*r (4).

In Section “Discussion” of this paper, the formula given above was expanded to add the contribution of the peritoneum and the strength of the fixation points to the resistance of the hernia repair toward DIS testing. The dimensionless number yielded from MDAR, peritoneal friction, and the strength of the fixation points is called grip since it represents the static friction between the hernia repair and the pig tissue.

### Study Design

The grip factor was assessed considering different meshes previously classified as DIS class A and B meshes with dynamic intermittent strain [DIS, Table 1 according to Ref. (9)]. The major aim was to find various durable repair techniques based on one comparative figure. For this purpose, three different meshes were included previously classified as DIS class A or B (9, 10). Parietex Progrip^®^ uses denticles to stabilize the mesh on the tissue surface. Dynamesh Cicat^®^ was especially designed with an anti-slip effect (12). The mesh was used as instructed by the manufacturer since a perpendicular orientation drastically alters the stickiness of the mesh (9). For Dynamesh IPOM^®^, fixation with four sutures is mandatory in the clinical setting. For experimental purposes, all meshes were tested without fixation in preliminary experiments to assess the DIS class the mesh belongs to Ref. (9, 10). Choosing primarily unstable conditions, stabilization is sought by various fixation techniques with tacks, sutures, and gluing spots. From 20 different reconstructions tested with 10 repetitions each, the grip factor for each reconstruction is calculated to obtain the threshold for safe repairs using a particular reconstruction. A comprehensive overview of the series conducted is given in Table 2. In this table, the grip is estimated from the results obtained after all series were completed. Peritoneal closure acted as an addend with the number of 4. Sutures, tacks, and glue were detected to act as a multiplier. Sutures, Securestrap^®^, and ProTack^®^ were found to increase fixation strength by 0.5 for each bonding point. AbsorbaTack^®^ and Glubran glue^®^ increased bonding as a factor of 0.33 per spot. The grip was calculated as

(1) $$\text{Grip}={\text{MDAR }}^{*}\text{ Bonding factor}+\text{Peritoneum factor}$$

**Table 1:** Proposal for a classification based on dynamic intermittent strain (DIS) testing, including previous cost calculation and pain assessment combined with a potential clinical use according to Ref. (9).

Classification	Application	Added cost	Clinical use	Patient comfort
Class A	Self-retaining: no or little fixation needed	<500 €	Hyperreactive bronchi COAD, Re-Do surgery, bony edges and scars etc.	Less pain, less recurrences
Class B	Needs half-maximal fixation	500–1,000 €	Coughs up to 150 cycles	Medium pain levels due to the fixation necessary
Class C	Needs best fixation available	> 1,000 €	Specialized, e.g., biomaterials	High pain due to the increased need for fixation

**Table 2:** Descriptive statistical parameters of the 20 series conducted on three different meshes.

Condition	Mesh	Hernia orifice (cm)	Overlap (OL) (cm)	Fixation	Mean	SE	Minimum	First Quartile	Median	Third Quartile	Maximum	Grip estimate
**Dynamesh CiCat^®^ sublay flat, with recommended and reduced OL and with peritoneum intact or with a transmural hernia orifice (Figure 2)**												
Peritoneal defect	Cicat^®^	5	5	None	425	0	425	425	425	425	425	9
Peritoneal defect	Cicat^®^	5	3.75	None	226	176	13	106	147	425	425	6.25
Peritoneal defect	Cicat^®^	5	2.5	None	17	9	8	8	14	23	29	4
Peritoneum intact	Cicat^®^	5	2.5	None	425	0	2	425	425	425	425	8.1
**Dynamesh CiCat^®^ sublay flat, with recommended and reduced OL of a constant hernia orifice without or with Glubran^®^ fixation (Figure 3)**												
No fixation	Cicat^®^	5	5	None	425	0	425	425	425	425	425	9
No fixation	Cicat^®^	5	3.75	None	216	164	11	108	135	425	425	6.25
4 spots Glubran^®^ (4PG)	Cicat^®^	5	3.75	4PG	425	0	425	425	425	425	425	8.1
8 spots Glubran^®^ (8PG)	Cicat^®^	5	1.25	8PG	80	94	12	16	38	109	305	5.9
8 spots Glubran^®^ (8PG) and 4 sutures (4S)	Cicat^®^	5	1.25	8PG and 4S	425	0	425	425	425	425	425	11.7
**Dynamesh CiCat^®^ and Parietex Progrip^®^ sublay flat, with reduced OL of a larger hernia orifice without or with fixation (Figure 4)**												
No fixation	Cicat^®^	7.5	3.75	None	1	1	1	1	1	1	1	4
8 sutures (8S)	Cicat^®^	7.5	3.75	8S	311	178	7	195	425	425	425	16
No fixation	Progrip^®^	7.5	3.75	None	230	206	1	8	299	425	425	4
8 sutures (8S)	Progrip^®^	7.5	3.75	8S	425	425	425	425	425	425	425	16
**Dynamesh IPOM^®^, bridging a larger hernia orifice with a reduced OL without and with 4, 8, and 12 fixation points (Figure 5)**												
No fixation	IPOM^®^	7.5	3.75	None	3	2	1	2	2	2	6	4
4 sutures (4S)	IPOM^®^	7.5	3.75	4 transmural	13	12	3	5	9	17.5	42	8
4S and 4 spots Glubran^®^ (4G)	IPOM^®^	7.5	3.75	4S and 4G	179	179	5	46	101	369	425	10.4
4S and 4 Securestrap^®^ tacks (4SS)	IPOM^®^	7.5	3.75	4S and 4 SS	302	198	5	121	425	425	425	16
4S and 4 Protack^®^ tacks (4PT)	IPOM^®^	7.5	3.75	4S and 4 PT	316	178	15	210	425	425	425	16
4S and 4 Absorbatacks^®^ (4AT)	IPOM^®^	7.5	3.75	4S and 4 AT	116	151	4	9	34	197	425	10.4
4S and 8 Securestrap^®^ tacks (4SS)	IPOM^®^	7.5	3.75	4S and 8 SS	425	0	425	425	425	425	425	24

Plotting the likelihood of any repair tested with 10 repetitions to survive more than 400 DIS impacts against the grip estimated from the factors given above, the absolute measure of the grip to be passed for a durable repair was estimated for each mesh. Care must be taken to transfer the results into clinical practice since the singular closure of the peritoneum without the closure of the muscular defect is completely unusual and not applicable in the clinical setting.

### Statistical Analysis

After starting the model, the mesh-tissue compound moved until the full circumference of the tissue defect was exposed or the delivery of 425 cycles of dynamic intermittent impacts were completed. The final count of the impacts delivered was noted from the LabView screen (8–10). The variability of the 425 DIS impacts was analyzed to measure the variability of the peak pressures. The variability was found to be below 4% (range: 180–250 mmHg; mean + SD: 204 + 14 mmHg).

The data acquired are typically skewed. Parametric and non-parametric data are given. For statistical analysis, Kruskal–Wallis and Mann–Whitney *U* tests were used as reported previously (9, 10). For graphical representation, box-and-whisker-plots were chosen. Since box-and-whisker-plots cannot discern small differences, likelihood curves of dislocation resembling survival curves were calculated from the acquired data and are provided additionally for clarification. Plotting the likelihood of any repair tested with 10 repetitions to survive more than 400 DIS impacts against the grip estimated from the factors given above, the absolute measure of the grip to be passed for a durable repair was obtained for each mesh.

## Results

### The Influence of Reduced OL and Increased Grip of the Peritoneum on Dynamesh CiCAT^®^ Bridging a Hernial Orifice with a Diameter of 5 cm

**Figure 2:**
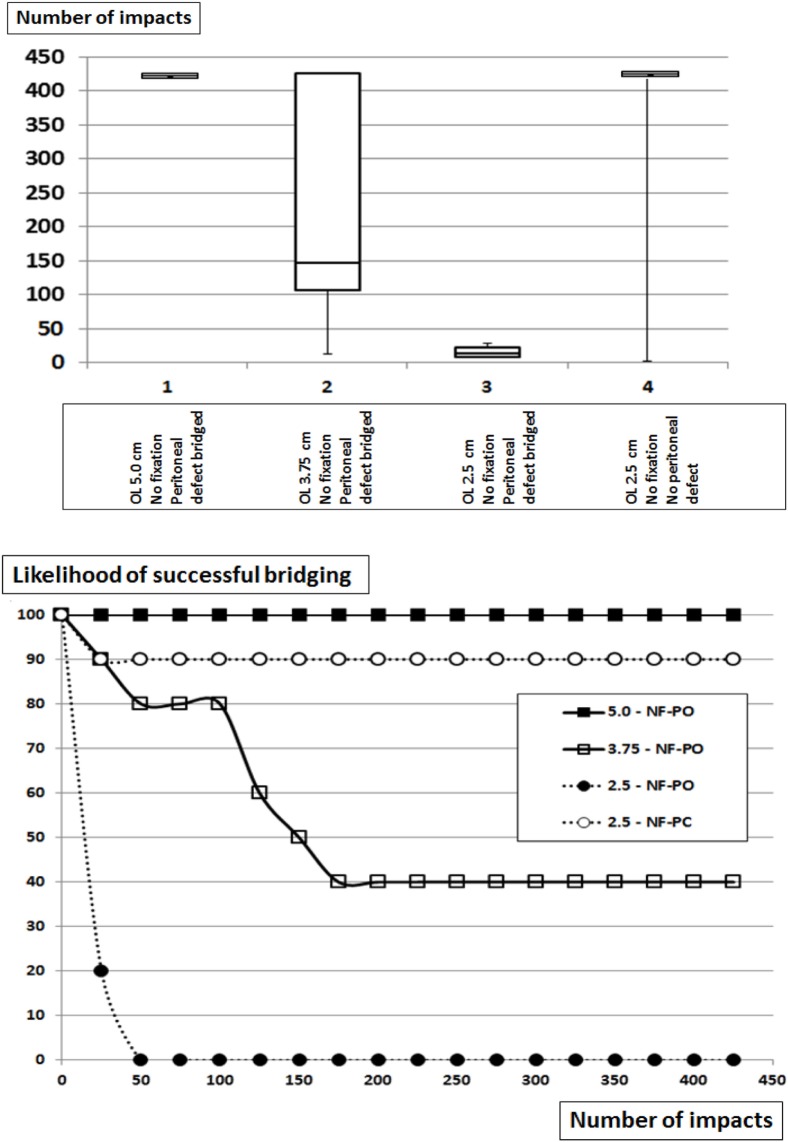
Top: box-and-whisker-plots of the cycles with complete dislocation for Dynamesh Cicat^®^ bridging a hernia orifice of 5 cm in a sublay position without fixation at reduced overlap. The peritoneum bears a hernia orifice (PO) of 5 cm or is left intact. Bottom: likelihood curves of the DIS tested hernia repairs using Dynamesh Cicat^®^ in a sublay position. The curves correspond to the conditions given in the box plots above. NF = without fixation.

Dynamesh CiCAT^®^ in a sublay position can safely bridge a hernia with a diameter of 5 cm using an OL of 5 cm and is, thus, classified as DIS class A (Figure 2; Table 2). Upon reduced OL the safety of the repair rapidly deteriorates. With an OL of 3.75 cm, the likelihood of a successful repair drops by 60–40% (statistically not significant). No repair withstands more than 30 impacts with an OL of 2.5 cm (*p* = 0.00018). Leaving the peritoneum intact almost brings the repair back to safety levels observed with the 5 cm OL with a 90% likelihood to withstand 425 DIS impacts. The grip factor is 9 with an OL of 5 cm, 6.25 with an OL of 3.75 cm and 4 with an OL of 2.5 cm bridging a hernial orifice of 5 cm with the peritoneum open. Since the intact peritoneum restores the likelihood of successful bridging from 0 to 90% a static friction similar to an additional 2 cm of OL or a bonding with four spots Glubran^®^ can be reached by closing the peritoneum. The grip necessary to prevent dislocation was 9 in this series. The assessment of this type of repair had the purpose to study grip changes *in vitro* since the method is not applicable in human surgery.

### The Influence of Reduced OL and Increasing Glubran^®^ Fixation on the Grip of Dynamesh CiCAT^®^ Bridging a Hernial Orifice with a Diameter of 5 cm

**Figure 3:**
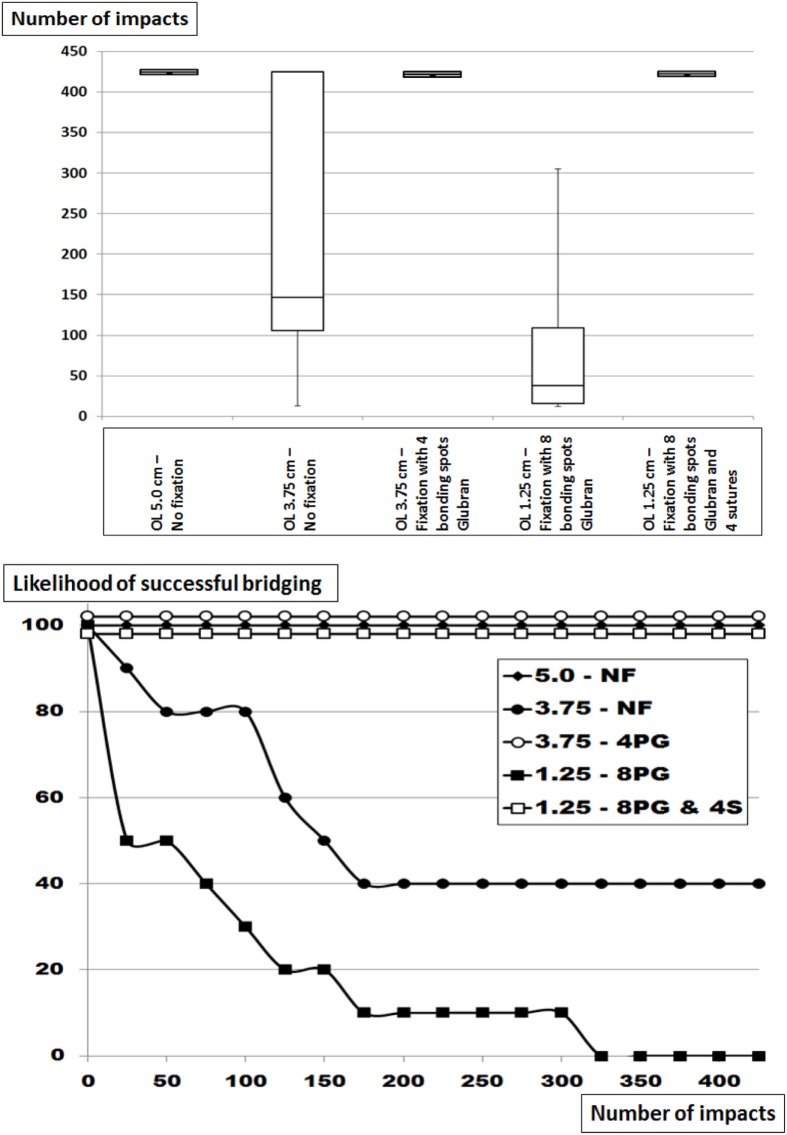
Top: box-and-whisker-plots of the cycles with complete dislocation for Dynamesh Cicat^®^ bridging a hernia orifice of 5 cm in a sublay position without fixation or with Glubran^®^ bonding spots with and without corner sutures at reduced overlap. The peritoneum bears a hernia orifice of 5 cm. Bottom: likelihood curves of the DIS tested hernia repairs using Dynamesh Cicat^®^ in a sublay position. The curves correspond to the conditions given in the box plots above. NF = without fixation, 4 or 8 = number of bonding spots, PG = bonding spots of Glubran^®^, 4S = 4 corner sutures.

In this series, Dynamesh CiCAT^®^ was again placed in a sublay position bridging a 5-cm hernia defect (Figure 3; Table 2). At reduced OL without fixation, earlier dislocation occurred similar to that observed above at comparable OL and more pronounced at lower OL (*p* = 0.00012). Using four bonding spots Glubran^®^, no dislocation was observed after repeating 425 DIS impacts in 10 different preparations (*p* = 0.0233) despite an OL reduction to 3.75 cm. Further reducing the OL to 1.25 cm increased the need to fixation to 12 spots in order to prevent dislocation. A combination of four transmural sutures and eight bonding spots Glubran were sufficient to achieve this aim (*p* = 00018). The grip necessary to prevent dislocation varied between 8.1 and 11.7 under these conditions.

### The Influence of Increasing Fixation on the Grip of Dynamesh CiCAT^®^ and Parietex Progrip^®^ Bridging a Hernial Orifice of 7.5 cm with an OL of 3.75 cm

**Figure 4:**
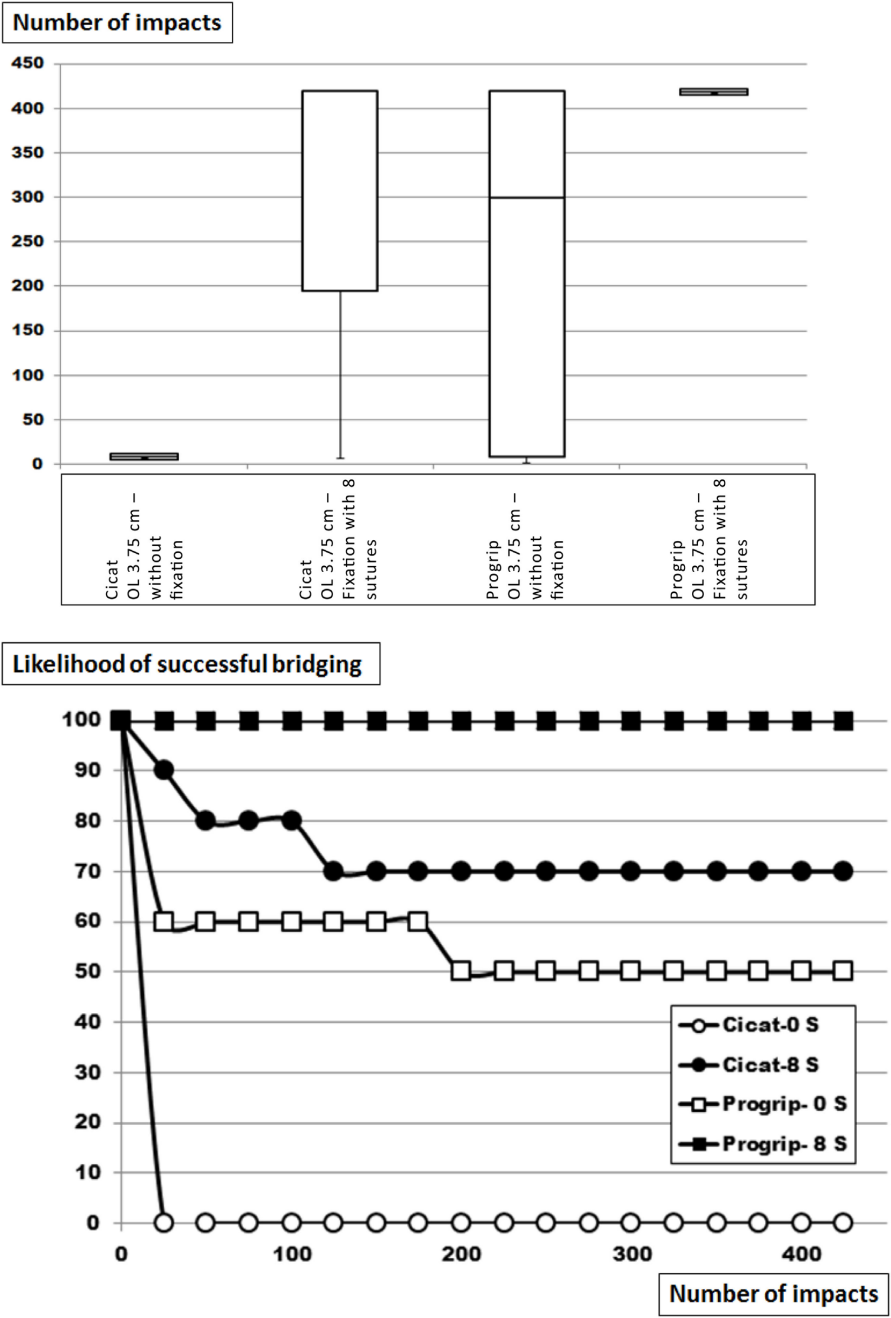
Top: box-and-whisker-plots of the cycles with complete dislocation for Dynamesh Cicat^®^ and Parietex Progrip^®^ bridging a hernia orifice of 7.5 cm in a sublay position without fixation or with eight evenly distributed transmural sutures at reduced overlap of 3.75 cm. The peritoneum bears a hernia orifice of 5 cm. Bottom: likelihood curves of the DIS tested hernia repairs using Dynamesh Cicat^®^ and Parietex Progrip^®^ in a sublay position. The curves correspond to the conditions given in the box plots above. S = number of sutures.

Dynamesh CiCAT^®^ and Parietex Progrip^®^ are both classified as DIS class A bridging a 5-cm hernia with an OL of 5 cm, but are unstable when bridging a hernia with a diameter of 7.5 cm with an OL of 3.75 cm (Figure 4; Table 2). Under these conditions, Progrip^®^ is more stable than CiCAT^®^ (*p* = 0.00078) with 50% of the Progrip^®^ reconstructions being in place after 425 DIS impacts. Retaining the meshes with eight sutures markedly elevates the fixation strength in both cases. Dynamesh Cicat^®^ stayed in place in 7 out of 10 experiments (*p* = 0.0018). Using Progrip^®^ with 8 sutures, no dislocation occurred in any of the 10 repetitions (n.s.). Without fixation, grip equaled MDAR and was 4. With 8 sutures, grip was elevated in both meshes up to 16.

### The Influence of Increasing Fixation on the Grip of Dynamesh IPOM^®^ Bridging a Hernial Orifice of 7.5 cm

**Figure 5:**
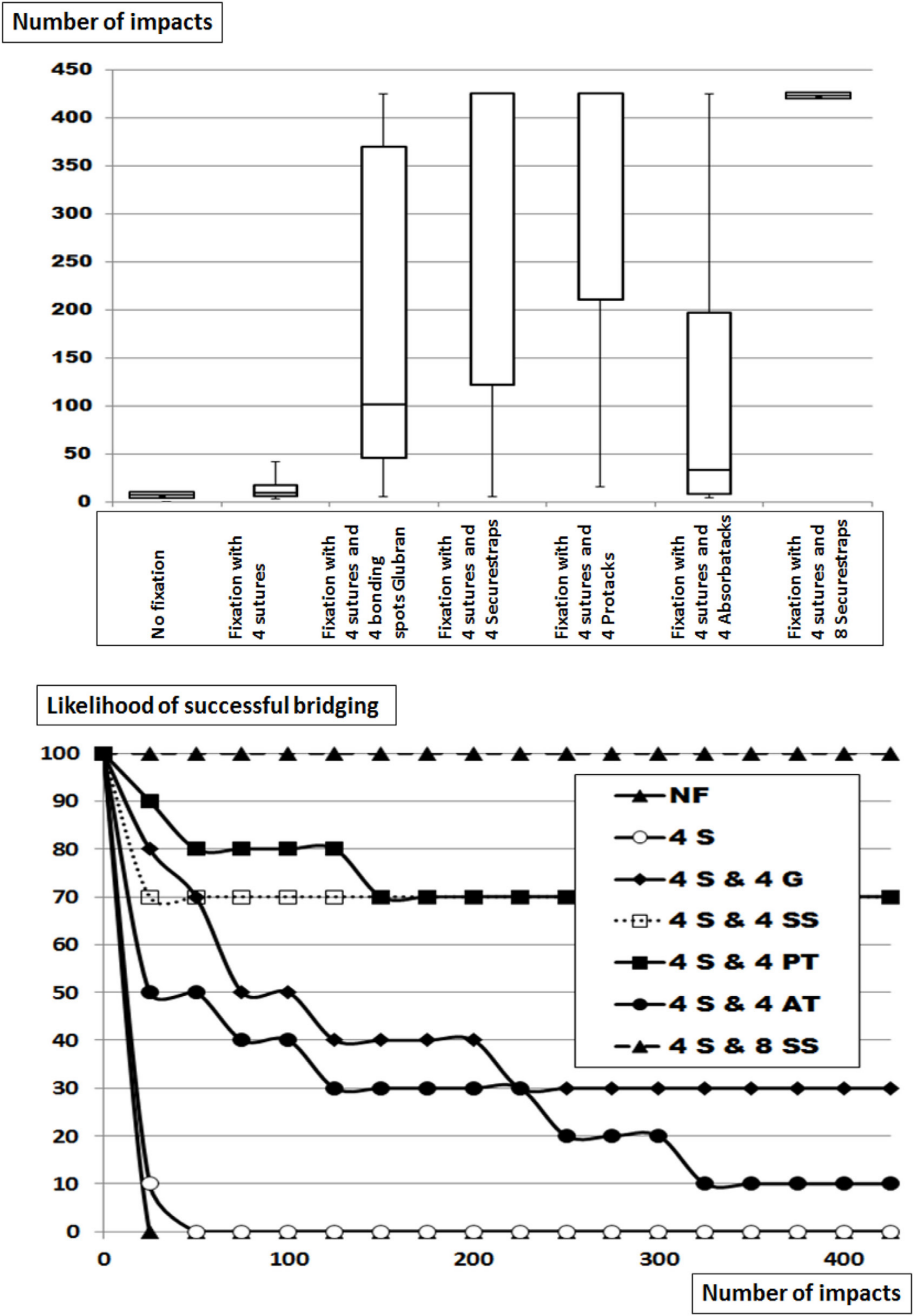
Top: box-and-whisker-plots of the cycles with complete dislocation for Dynamesh IPOM^®^ bridging a hernia orifice of 7.5 cm in an underlay position without fixation, with corner sutures alone (S) or combined with four bonding spots Glubran^®^ (G), four or eight Securestrap^®^ tacks (SS), or with four ProTacks^®^ (PT) or four AbsorbaTacks^®^ (AT) at an overlap (OL) of 3.75 cm. The peritoneum bears a hernia orifice of 5 cm. Bottom: likelihood curves of the DIS tested hernia repairs using Dynamesh IPOM^®^ in an underlay position. The curves correspond to the conditions given in the box plots above. NF = without fixation.

Dynamesh IPOM^®^ is recommended by the manufacturer to be secured with minimally four corner stitches (Figure 5; Table 2). For DIS classification, the mesh was tested without fixation to bridge a 7.5-cm defect with a 15-cm round mesh. Placing the mesh with four corner stitches significantly increased stability (*p* = 0.00132) but no reconstruction survived 425 DIS impacts. Using additional fixation, two distinct patterns were discerned: with four points Glubran^®^ or with four AbsorbaTacks^®^, weak fixation was achieved with less than 50% of the reconstructions surviving 425 DIS impacts the increase in stability not being significant. Adding Securestrap^®^ or ProTack^®^, safety levels of 70% were reached (*p* = 0.00672 and *p* = 0.00058, resp). Using 12 point fixation with four sutures and eight Securestraps^®^, the 100% safety level was reached again (*p* = 0.00018).

Without fixation, the grip was derived from the MDAR being 4. With 4 sutures, grip doubled to 8. With 4 additional weak fixation spots (Glubran^®^ and AbsorbaTack^®^), grip was increased slightly above 10. With 4 additional strong fixating bonds (Securestrap^®^ and ProTack^®^), grip increased to 16. All reconstructions survived 425 DIS impacts with 12 strong fixation points, the grip being 24 with 4 sutures and 8 Securestraps^®^.

### Relationships between the Calculated Grip and the Likelihood to Withstand 425 DIS Impacts

**Figure 6:**
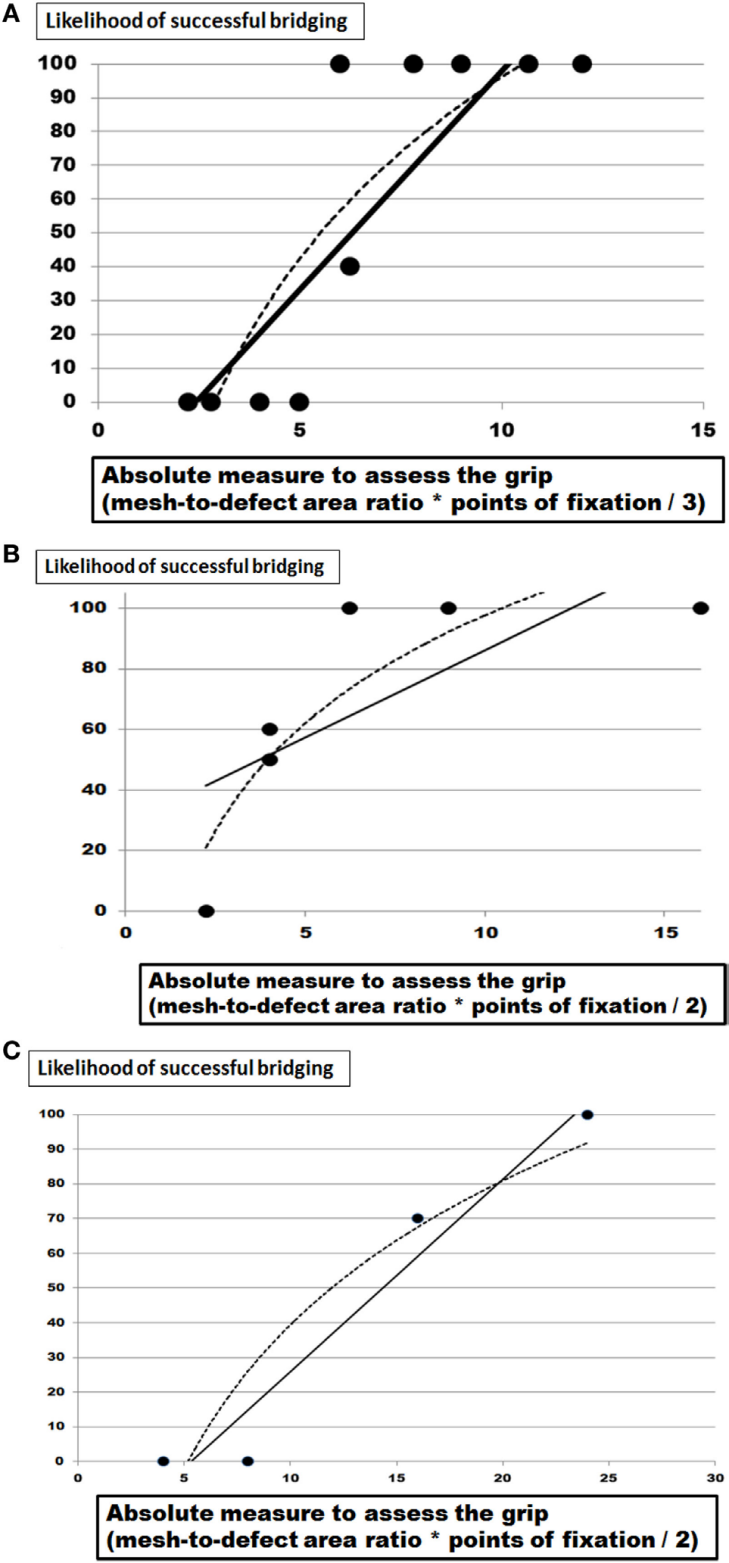
**(A)** Plot of the likelihood of successful bridging a 5 or 7.5 cm hernia orifice after 425 DIS impact using Dynamesh Cicat^®^ with various fixation devices and overlap (OL) areas as given in Figures 2–4 as a function of the grip calculated as (1) given in the text. **(B)** Plot of the likelihood of successful bridging a 5 or 7.5 cm hernia orifice after 425 DIS impact using Parietex Progrip^®^ with various fixation devices and OL areas as given in Figure 4 and in Ref. (9, 10) related to the grip calculated as (1) given in the text. **(C)** Plot of the likelihood of successful bridging a 5 or 7.5 cm hernia orifice after 425 DIS impact using Dynamesh IPOM^®^ with various fixation devices and OL areas as given in Figure 5 and in Ref. (9, 10) related to the grip calculated as (1) given in the text. The solid line gives a linear fit and the dashed one represents a logarithmic trendline.

Plotting the likelihood of any repair tested with 10 repetitions to survive 425 DIS impacts against the grip estimated from the factors given above, the absolute measure of the grip to be passed for a durable repair was obtained for each reconstruction with more than three experimental series including previously published data for Dynamesh Cicat^®^ and Progrip^®^ (10). The results are depicted in Figure 6.

For the three meshes investigated here, a similar pattern is obvious. Starting at low grip levels, dislocation occurs frequently. At higher grip values, 100% likelihood for the reconstruction to survive 425 DIS impacts is reached. For Dynamesh Cicat^®^ fixated with Glubran^®^ bonding spots, the desired 100% level is reached with a grip 7 (Figure 6A). For Progrip^®^ stitched with sutures or tacked with Securestrap^®^, the desired 100% level is again reached with a grip of 7 (Figure 6B). Since both meshes are DIS class A meshes, low grip values are expected. For Dynamesh IPOM^®^, fixed with strong attachment devices such as transmural sutures or strong tacks (Securestrap^®^ or ProTack^®^), a grip value of 24 is needed to avoid dislocation upon 425 DIS impacts (Figure 6C). Analyzing the best fitting curve in more detail, it cannot be decided whether a linear, logarithmic, or polynomic fit might be more generally applicable in the future.

## Discussion

Defect closure is still debated in ventral hernia repair and is sought after even during laparoscopic or robotic surgery (13, 14). The data presented provide evidence for a biomechanical contribution of the peritoneum to stabilize ventral hernia repair (Table 2; Figure 2). It is consented that the fascia should be closed as long as reasonable possible (15, 16). The role of the peritoneum is debated at the moment either being omittable or being a rescue technique. Recent reviews found no evidence for any short-term or long-term advantage in peritoneal closure (17, 18). By contrast, Malik et al. (19) reported a technique based on a peritoneal flap. From a biomechanical viewpoint, a peritoneal reinforcement can give a grip similar to four bounding spots Glubran^®^. The study conducted here assessed grip *in vitro* and caution has to be exerted to transfer the results to the clinical situation. A prospective observation was started adding MDAR and grip to the HERNIAMED registry for participating centers in order to gain more insight into the recurrence rates related to MDAR and grip.

Glubran^®^ bonding was found to be comparable to AbsorbaTack^®^ being a weak fixation device (Table 2; Figures 2–4). This was in contrast to the various transmural sutures used, Securestrap^®^ and ProTack^®^ as strong fixations. Sutures were in our hands exchangeable in the size ranges used here. Mechanical evaluation of Absorbatack^®^, Protack^®^, and Securestrap^®^ in a shear mode with testing conducted in foam commonly used to evaluate tack performance showed the ProTack^®^ fixation device to be three times stronger than Securestrap^®^ fixation device in this bench top test (*p* < 0.001 (20)). Using pull-out test with staples fixing four different meshes, we found the mesh orientation and its pore sizes as well the application pressure, the angle and the orientation of the tacks to influence the resistance to pull-out with a Minizwick^®^ (Zwick Roell, Ulm, Germany). Comparing pull-out forces limited by a titanium-coated mesh, the pull-out strength varied fivefold, being 2.6 ± 2.6 N using AbsorbaTack^®^, 10.81 ± 5.8 N with Securestrap^®^ and 13.7 ± 0.9 N with ProTack^®^ (*p* < 0.05). Comparing the pull-out forces limited by the tissue only, the pull-out strength varied twofold, being 11.2 ± 5.8 N for AbsorbaTack^®^, 12.4 ± 2.4 N for Securestrap^®^ and 22.7 ± 5.1 N for ProTack^®^ (*p* < 0.01). Since pull-out tests are performed with slow motion, dynamic impact DIS testing is superior to assess the interactions between fixation methods, tissues, and meshes. DIS testing could assess obvious differences between various fixation techniques (21, 22). *In vitro* testing permits the assessment of grip as a biomechanical aspect independent from the clinical situation. Pore size preventing fixation of certain meshes with given tackers and off-label-use of Glubran^®^ are two aspects of the transfer problem.

**Figure 7:**
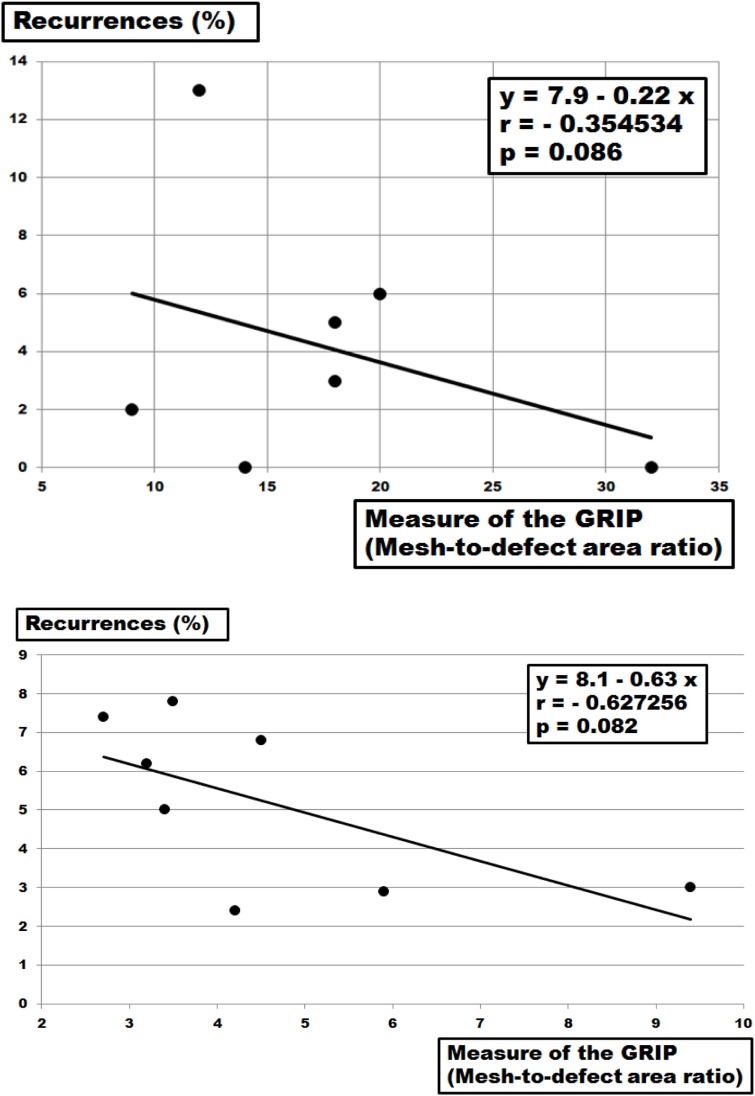
Top: plot of the recurrence rates of clinical cohorts as given by Tse et al. (5) with various fixation devices and overlap areas bridging hernia diameters up to 10 cm as a function of the grip calculated as mesh–defect area ratio (MDAR). The solid line gives a linear fit. Bottom: plot of the recurrence rates of clinical cohorts as reviewed by LeBlanc (3) with various fixation devices and (OL) areas bridging hernia diameters up to 10 cm as a function of the grip calculated as MDAR. The solid line gives a linear fit.

Reduced OL is detrimental at least in laparoscopic ventral hernia repair (3). In our series, reduced OL was used to destabilize primarily stable repairs and to assess the relative performance of fixation methods (Table 2; Figures 2–5). Using slow pushing or bending forces, an OL of 5 cm is sufficient for most meshes to successfully bridge a 5-cm hernial orifice (23). In clinical data, MDAR rather than OL was found to markedly influence recurrence (4–6). Plotting the data available for recurrences over MDAR, recurrences drop both in subgroups and in review accumulations [Figure 7; Ref. (3, 5)]. The grip factor is directly related to the area of the mesh and to the diameter of the hernia orifice. According to van’t Riet et al. (24), a maximum of seven sutures should be necessary to securely fasten a hernia mesh to bridge a 5-cm hernial orifice in pig tissue. Since this work was done on tissue stripes pulling only in one direction, circumferential strain either biplanar or ball-related might give different results (25). Since DIS testing uses circumferential strain the relative contribution of the fixation can be added to MDAR.

**Figure 8:**
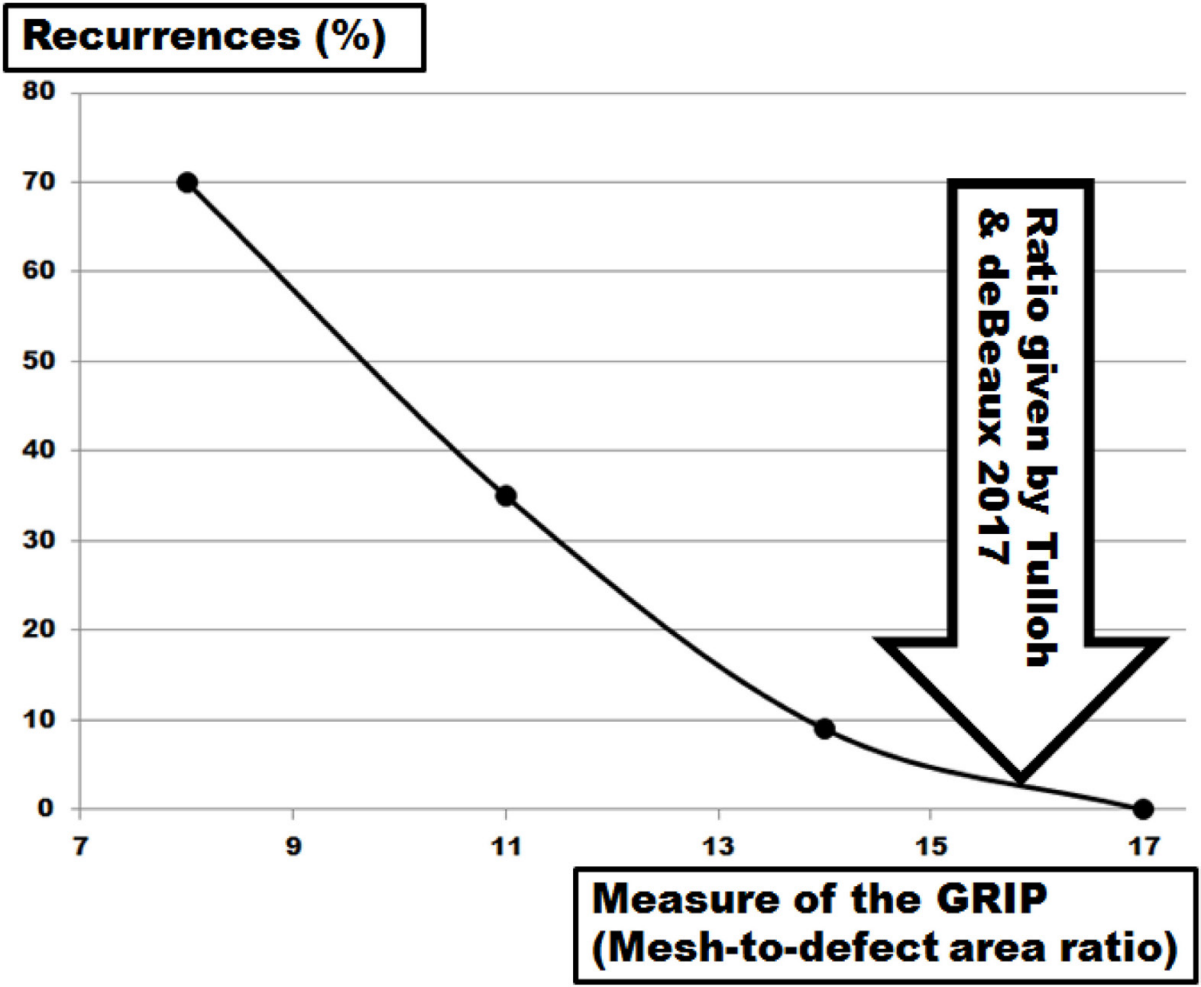
Plot of the recurrence rates of clinical cohorts as given by Hauters et al. (6) with various fixation devices and overlap areas bridging hernia diameters up to 10 cm as a function of the grip calculated as mesh–defect area ratio. The solid line gives a trendline. The arrow indicates the threshold for a ventral hernia repair with low recurrence (4).

Grip varies widely related to meshes, tissues, and fixation used due to the different biomechanical properties of each component (22, 26, 27). Calculating grip values from MDAR only turns out numbers within the range of the figures observed in this study, namely 4–30. Reconstructions surviving more than 425 DIS impacts require 9–11 for DIS class A meshes and above 24 for the DIS class B mesh studied here. From the data given by Hauters et al. (6) and recalculating the data from Tse et al. (5) and LeBlanc (3), the number of 16 seems to yield a recurrence rate below 3% after several years of observation (Figures 7 and 8). Searching for a comparable low figure, the MILOS technique has to be mentioned (28). Since a small incision, an intact peritoneum, a sublay technique, a DIS class A mesh, and four corner stitches are combined with an closed fascia and a wide OL, a grip far above 16 can be usually calculated for this innovative ventral hernia repair. Based on these experimental and clinical data, MDAR or the grip concept can guide future developments in ventral hernia repair.

In the clinical registry in Germany (Herniamed), a section was added called STRONGHOLD in which participating centers can contribute data to calculate MDAR and grip and observe a patient cohort prospectively. Progress in DIS testing is monthly updated in the weblog www.hernia-today.com. The grip concept provides a biomechanical basis to understand better how reconstructions are to be kept in place until healing occurs. Caution must be exerted to transfer the preliminary data to clinical work since important influences, e.g., closure of the fascia, cannot be summarized yet. The data available were extracted for the STRONGHOLD section of HERNIAMED which is open for further centers to participate.

## Conclusion

Based on 20 different reconstructions with three meshes commonly used for ventral hernia repair, a dimensionless number called grip is calculated which can be modulated by fixation in a reproducible way. Higher grip values can improve the durability of ventral hernia repair.

## Ethics Statement

I hereby certify that the procedures and the experiments I have conducted respect the ethical standards in the Helsinki Declaration of 1975, as revised in 2000, as well as the national law as stated in art. 23 (EG) 1069/2009 with permit DE 08 221 1018 21. Experiments with laboratory animals were not conducted.

## Author Contributions

FK designed the study and milestoned its conduct. Acquisition of data was conducted by DG, FH, RN, RR, TS, MV, and FK. Analysis and interpretation of data was the responsibility of FH, RN and FK Drafting of the manuscript was done by RN and FK. The manuscript was critically revised by all authors.

## Conflict of Interest Statement

The authors declare that the research was conducted in the absence of any commercial or financial relationships that could be construed as a potential conflict of interest.
